# Ferromagnetic domain walls as spin wave filters and the interplay between domain walls and spin waves

**DOI:** 10.1038/s41598-018-22272-2

**Published:** 2018-03-02

**Authors:** Liang-Juan Chang, Yen-Fu Liu, Ming-Yi Kao, Li-Zai Tsai, Jun-Zhi Liang, Shang-Fan Lee

**Affiliations:** 1 Institute of Physics, Academia Sinica, Taipei, 11529 Taiwan; 2 Department of Physics, Fu Jen Catholic University, Taipei, 24205 Taiwan

## Abstract

Spin waves (SW) are low energy excitations of magnetization in magnetic materials. In the promising field of magnonics, fundamental SW modes, magnons, are accessible in magnetic nanostructure waveguides and carry information. The SW propagates in both metals and insulators via magnetization dynamics. Energy dissipation through damping can be low compared to the Joule heating in conventional circuits. We performed simulations in a quasi-one-dimensional ferromagnetic strip and found that the transmission of the propagating SW across the domain wall (DW) depends strongly on the tilt angle of the magnetization at low frequencies. When the SW amplitude is large, the magnetization tilt angle inside the DW changes due to the effective fields. The SW transmission, the DW motion, and the magnetization tilt angle couple to each other, which results in complex DW motion and SW transmission. Both SW filtering and DW motions are key ingredients in magnonics.

## Introduction

Spintronics, which employs the electron’s spin as a degree of freedom^1^, is a rapidly expanding field. The magnetic materials, whose magnetizations are a macroscopic manifestation of the electrons’ spin, have been utilized in magnetic field sensors and information storage media for decades. To define the magnetic states at nanoscales, the spin transfer torque^2,3^ or spin orbit torque effects^4^ are preferred to the external magnetic field due to the scaling issue. The drawback is the large current density, which has been required to date, and the Joule heating associated with it. Since spin waves (SW) can propagate in both metals and insulators, high-frequency device components can be designed to operate in various materials in the order of a one GHz to several hundred GHz regime, with low-energy dissipation, which can be achieved by reducing the Joule heating due to charge current. Several spintronic logic^5^ and magnetic memory devices^6,7^ have been proposed. The generation and manipulation of the SW propagation in both ferromagnetic and antiferromagnetic materials have been intensively investigated recently in view of both fundamental research and the potential for technological applications^8–23^. Interests include the basic wave properties and characteristics, passive components in magnonic circuits, interferometers^14,21^, amplifiers^12^, and transistors^9^, etc. In some applications, moving the domain wall (DW) in a controlled manner is an important issue. Many research groups have proposed that the propagating SWs in a ferromagnetic nanowire are able to assist magnetic DW motion^24–31^. SWs can drive the DW effectively since they convey magnonic spin current. Magnons can be considered as spin-1 bosons with both angular momentum ±*ħ* and linear momentum *ħ***k**^25,30^. It has been theoretically shown that magnonic spin-transfer torque (STT) causes a DW to propagate in the direction opposite to the SWs^25^, and that the linear momentum transfer from magnons causes a DW to propagate in the direction of SWs^27^. The former occurs in one-dimensional (1D) systems, where the SW propagation can be described by a Schrödinger equation with a special potential term such that the SW has to transmit through a DW^25^. The latter occurs when the SWs are reflected by DWs in 2D nanostrips^26–29^. In perpendicular magnetic anisotropy (PMA) nanostrips^27,28^, the SW can be classified as the magnetostatic forward volume wave in the traditional definition based on the direction of the SW propagation relative to the magnetization of the PMA materials. Magnetic wires with in-plane magnetizations, thus the SW can be classified as backward volume wave, were also discussed^25,26,29^. However, the restricted width dimension acts as a high pass filter. Whether the dipolar SW can propagate needs careful investigation. High frequency propagating waves revealed the exchange SW dispersion relation in nature^29^. When the SW passes through a DW, the magnonic spin current changes its sign. As a result, there is a spin angular momentum transfer from the propagating magnons to the DW, which generates a torque and induces the DW motion opposite to the SW to absorb this torque. When the SW is partially or completely reflected^27,28^, the linear momentum transfer of the SW reflected at the magnetic DW induces an effective field which results in the rotation of the DW magnetization plane and forward motion of the DW^28,31^. Interactions between the SW and the DW are related to the geometry and dimensions of the magnetic elements, and their material parameters, such as the magnetic anisotropy constant and the saturation magnetization. However, in contrast to the cases of DW motion induced by polarized charge current, the SW-driven DW motion is still under development^30^.

We show here by simulation that the transmission ratio of the SW amplitude depends on the DW orientation in PMA nanostrips. Furthermore, the dispersion of DW motion is much more complex due to the rotation of the magnetization inside the DW. A simple 1D model, which describes analytically the transmission ratio of the SW through the DW, self-consistently checks the DW motion and fits the simulation results qualitatively well.

## Results and Discussion

**Figure 1:**
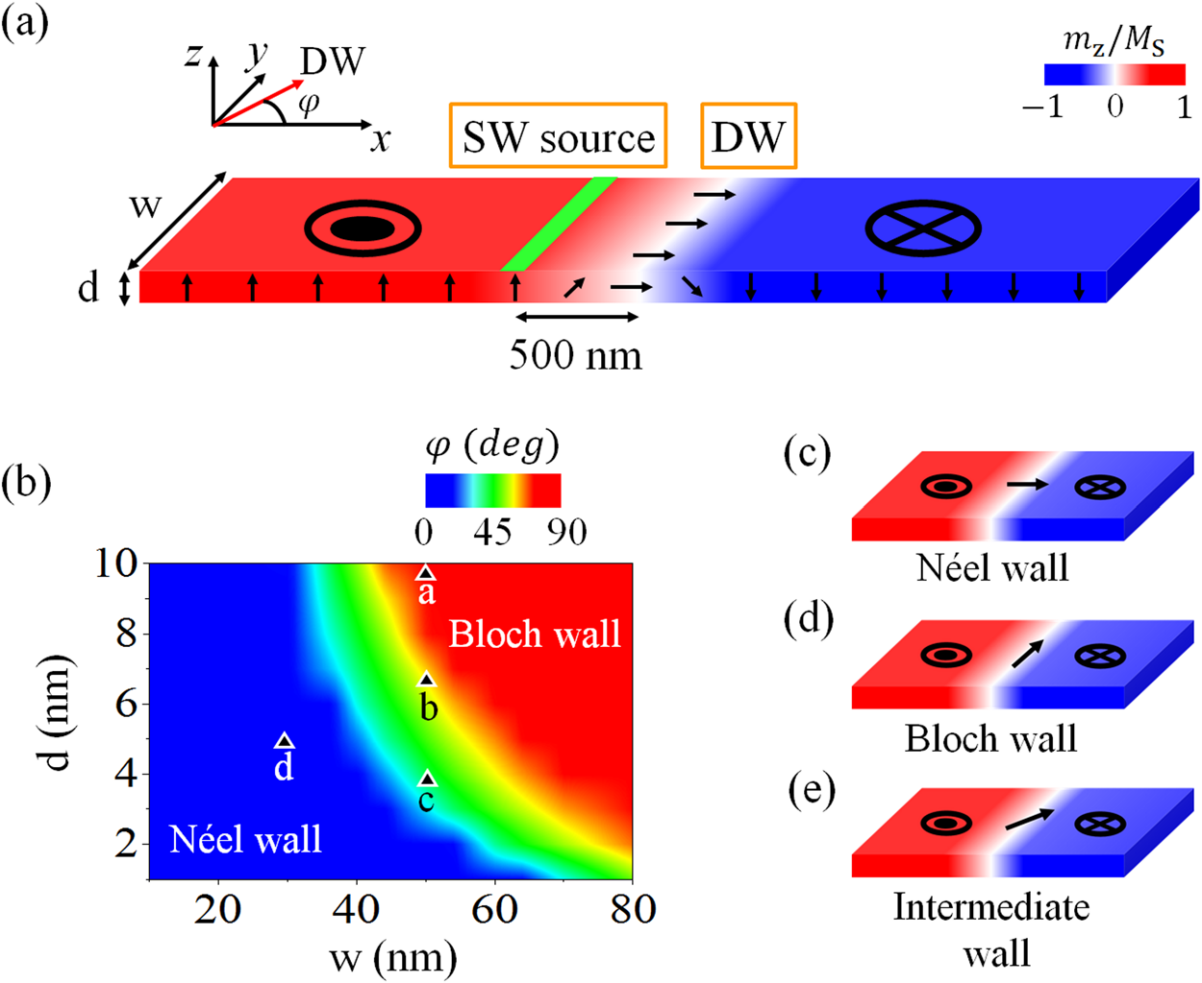
(**a**)Schematics of the sample geometry for simulation. The nanostrip is 4 µm long, with a domain wall located at the center, and a spin wave source 0.5 µm to the left. (**b**) Phase diagram of the equilibrium DW structure for width w ranging from 10 to 80 nm and thickness d from 1 to 10 nm. The structures of the DW correspond to (**c**) the Néel wall (center magnetization azimuthal angle φ = 0 or 180° to the x axis in the xy plane), (**d**) the Bloch wall (φ = 90 or 270°), and (**e**) an intermediate wall.

Our simulation setup is depicted in Fig. 1(a). A PMA nanostrip of width w, and thickness d is considered. Figure 1(b) shows the phase diagram of the equilibrium DW structure for w ranging from 10 to 80 nm, and d from 1 to 10 nm. The structures of the DW corresponding to the Néel wall (center magnetization azimuthal angle φ = 0° or 180° to the x axis in the xy plane), the Bloch wall (φ = 90° or 270°), and the intermediate wall are shown in Fig. 1(c) to (e), respectively. The Néel walls occur for thin and narrow strips and the Bloch walls for thick and wide strips^32^. The transition from one wall type to the other is shown by the green area as intermediate walls.

The nanostrip serves as a waveguide for the SW with a cutoff frequency (*f*_c_), below which no SW can propagate, as determined either from simulation or by the analytical form of the dispersion relation^15,30^. In our case, *f*_c_ is around 14.3 GHz.

### Anisotropic transmission of small amplitude spin wave through static domain walls

**Figure 2:**
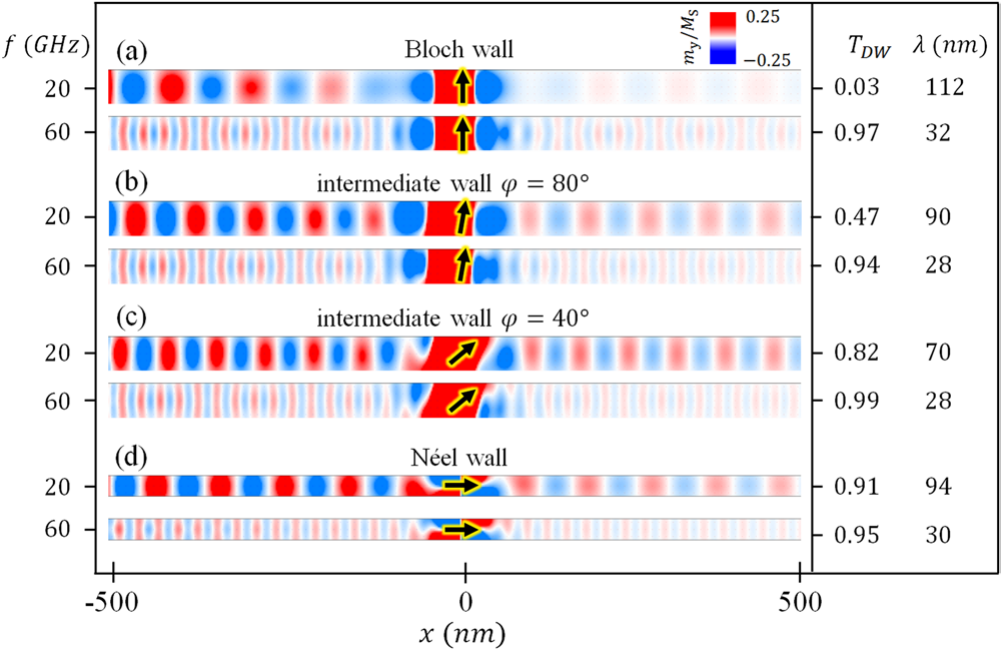
Top view of the 20 and 60 GHz SW transmission through different types of DW indicated in Fig. 1(b). Snapshots of the y component of the magnetization are shown. The black arrow indicate the orientation at the center of the DW. The transmission ratio and the respective SW wavelengths are shown on the right.

The transmitted amplitude of the SW passing through different types of DWs is summarized in Fig. 2. The incoming SW with frequencies of 20 and 60 GHz are considered for low and high frequencies. The excited field amplitude is ${H}_{0}/{M}_{S}=9.25\times {10}^{-3}$. Such a weak field is necessary in order to avoid the SW-driven DW motion. Note the SW transmission ratio, *T*_DW_, we define here is the SW amplitude ratio with and without a DW on the +x side. The SW amplitude ratio after and before the DW is not an issue here. In Fig. 2(a), when the 20 GHz SW passes across the Bloch wall, the amplitude is approximately null. When the SW with a larger frequency of 60 GHz passes the same wall, the amplitude is large and *T*_DW_ = 0.97. In the region of intermediate walls, the SWs with $f=20\,{\rm{GHz}}$ are partially transmitted. The *T*_DW_ = 0.47 and 0.82 corresponding to φ = 80° and 40°, respectively [Fig. 2(b) and (c)]. The transmission in these cases remains high. For the Néel wall, the SW almost transmits completely through the wall with both high and low frequencies as shown in Fig. 2(d). The wall width is about 16 nm for each of the different structures of DWs in our simulations, as can be seen in Fig. 2. All wavelengths we consider here are larger than the wall widths. We have found the transmitted amplitude of the SW passing through the DW is strongly dependent on the tilt angle of the DW at relatively low frequencies. We have worked out a 1D model, in which the SW has different attenuation lengths inside and outside the DW as shown below with details in the supplementary materials. The transmission ratio is a function of the DW width and attenuation lengths, which in turn are functions of SW frequency, geometry, and demagnetization factors, etc. These physical properties determine the DW structures as well as the *T*_DW_ of the SW in magnetic nanostrips.

### Spin wave induced domain wall motion and the resultant transmission

We studied how the propagation of the SW with a large amplitude changes the orientation of the DW’s magnetization and the dynamic of the DW motion. At high SW frequencies, the transmission is strong and the DW moves backwards, consistent with previous reports^25–28^. At low frequencies when the SW has a large amplitude, we obtained interesting results.

The dependence on DW width and SW frequency (wavelength) of the SW propagation and the DW motion were previously investigated by simulation in a PMA strip with a Bloch DW^27^. For large anisotropy or a narrow DW, complete transmission of the SWs in the high-frequency range was found. For small anisotropy or a wide DW, the complete transmission of SWs was extended to lower frequencies, even close to the cutoff frequency. The authors proposed that a dynamic stray field, which arises when a SW travels in a DW causing surface magnetic charges to appear on the DW boundaries, was the reason for the reflection of SWs. This field was approximated using the demagnetization factor ${{\rm{N}}}_{{\rm{DW}}}^{{\rm{X}}}$, which was determined by the DW width, ${\rm{\Delta }}={\rm{\pi }}{\sqrt{{\rm{A}}/{\rm{K}}}}_{\perp }$, where A is the exchange stiffness, and *K*_⊥_ is the perpendicular anisotropy constant^28^. The reflectivity of SWs was manipulated by changing the anisotropy constant, and hence the DW width. Although the dependence of DW motion on DW width and SW frequency has been investigated, the DW orientation has not been considered at length. It is necessary to release the constraint of rigid DWs and study how SW propagation and DW motion depend on the DW’s internal structures.

**Figure 3:**
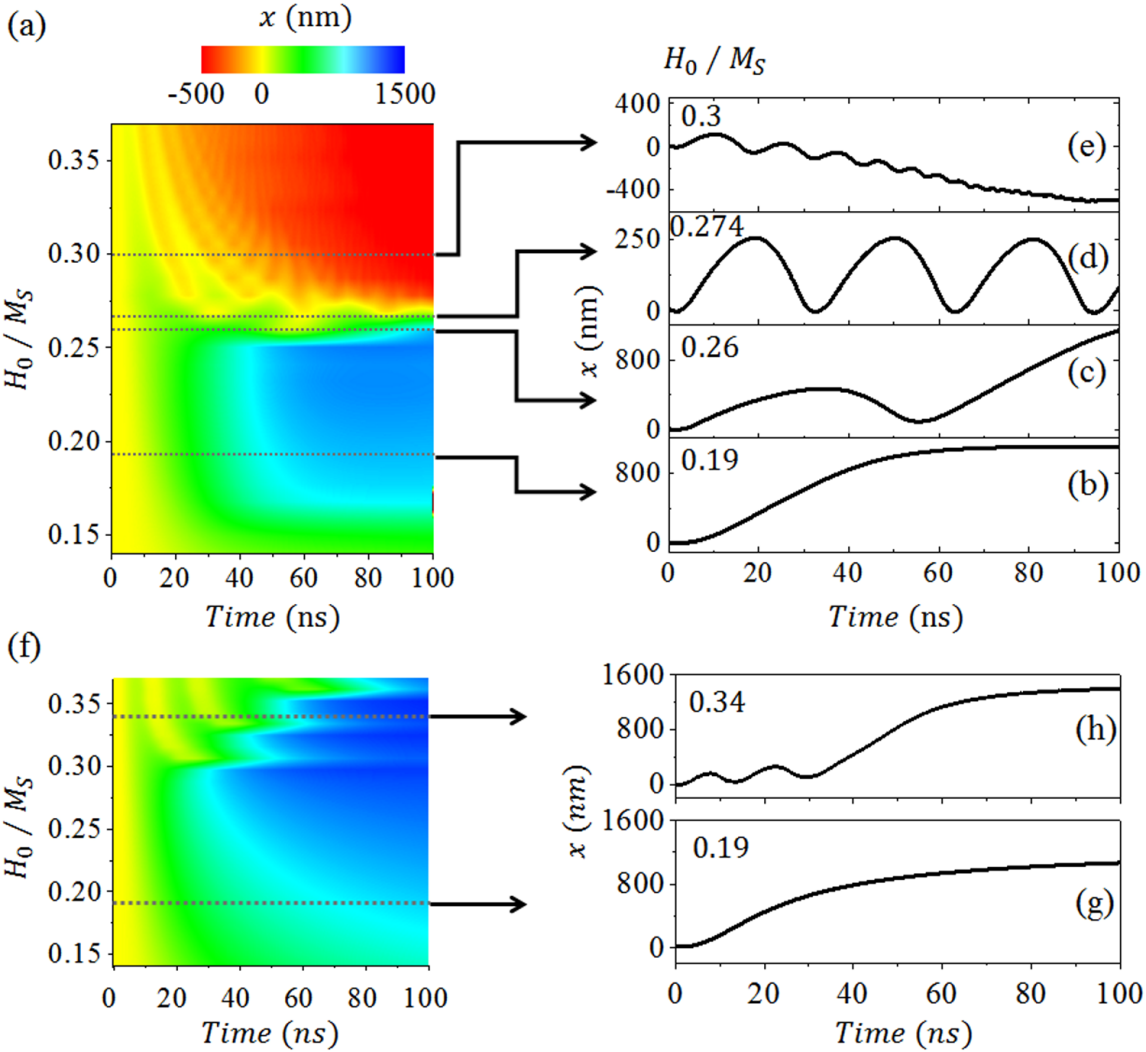
Typical domain wall displacement phase diagram as functions of simulation time and magnetic field amplitude of the SW with 20 GHz frequency. (**a**) for a Néel wall. (**b**) to (**e**), selected fields showing different types of domain wall motion. (**f**) phase diagram for a Bloch wall. (**g**) and (**h**), selected fields showing different types of domain wall motion.

Our results on the time evolution of the DW motion driven by SWs are shown in Fig. 3 (a) to (e) for a Néel wall and (f) to (h) for a Bloch wall with *f* = 20 GHz. Based on the characteristic profiles of the DW displacement versus time curves, the Néel wall motion induced by the SW can be separated into three regions with increasing SW amplitude. They are forward, oscillatory, and backward motions. We plotted the profile of dynamic DW motion with the displacement x as a two-dimensional phase diagram of *H*_0_ and *t*, as shown in Fig. 3(a). As we describe in the following paragraphs, when the SW is propagating through the DW, an effective field is induced resulting in magnetization rotation in addition to the motion of the DW. The transmission of a SW is dependent on the DW orientation that in turn influences the DW motion. By these interactions between the SW and the DW, the DW motion could become very complex.

For region I, forward displacement was obtained in the wide range with a small excitation amplitude as shown in Fig. 3(b) for *H*_0_*/M*_*S*_ = 0.19. Region II is very narrow in the phase space, 0.256 ≤ *H*_0_*/M*_*S*_ < 0.26, where the DW shows backward motion, though the final displacement is forward, as shown in Fig. 3(c) for *H*_0_*/M*_*S*_ = 0.26. Figure 3(d) shows the localized steady state oscillatory motion of the DW for *H*_0_*/M*_*S*_ = 0.274, corresponding to the boundary of region II and region III. The amplitude of this oscillatory motion is 140 nm and the period is 32 ns. In region III, the shape of the x versus t curve shows oscillatory motions of the DW associated with propagations in the opposite direction to the SW as shown in Fig. 3(e) with *H*_0_*/M*_*S*_ = 0.3. The DW acquires a negative average velocity, moves backwards, and is finally trapped at the SW source. In this situation, magnonic STT plays a crucial role in SW-induced DW motion. For larger *H*_0_, the structure of the DW is destroyed by the larger amplitude of the SW, and the rigid DW approximation is no longer valid. The total demagnetization torque vanishes due to the irregular structure of the DW.

The Bloch wall motion induced by the SW shows only two regions, and forward motion with or without oscillation, as shown in Fig. 3(f) to (h), at low frequencies. When *H*_0_*/M*_*S*_ = 0.19, the DW moves forwards as shown in Fig. 3(g). When the amplitude is increased, the DW motion starts to show oscillations, as shown in Fig. (h) for *H*_0_*/M*_*S*_ = 0.34.

We have found that the propagating SW can drive a DW motion in a manner depending on the in-plane rotation of the magnetization at the center of the DW. The instantaneous velocity of the DW is associated with changes of the azimuthal angle, *δφ*, in the DW structure. The average velocity depends on the transmission ratio, which is a function of *φ*.

### Instantaneous velocity as a function of *δφ*

The rotation of the DW plane plays a crucial role in the DW dynamics. The dynamics of the local magnetization when the SW is present is described by the modified Landau-Lifshitz-Gilbert (LLG) equation^27^,

1 $$\frac{\partial {\boldsymbol{M}}}{\partial t}=-\gamma {\boldsymbol{M}}\times {{\boldsymbol{H}}}_{{\rm{eff}}}+\frac{\alpha }{{M}_{{\rm{s}}}}{\boldsymbol{M}}\times \frac{\partial {\boldsymbol{M}}}{\partial t}-\frac{\partial {{\boldsymbol{J}}}_{{\rm{m}}}}{\partial x},$$

where *γ* is the gyromagnetic ratio, *α* is the Gilbert damping parameter, *M*_s_ is the saturation magnetization, ***H***_eff_ is the effective magnetic field consisting of anisotropy, demagnetization, and exchange fields, and ***J***_m_ is the magnon spin current. Notice that equation (1) describes the time dependent behavior of the magnetization, whereas the long-range magnetization correlation describes the SW propagation. Though the z-component of the magnetization across the DW is antisymmetric with respect to the wall center and can be described by m_z_(x) ∝ –arctan(x), the propagating SW has a decaying amplitude; thus, the magnonic spin torque breaks the antisymmetric structure of the DW.

**Figure 4:**
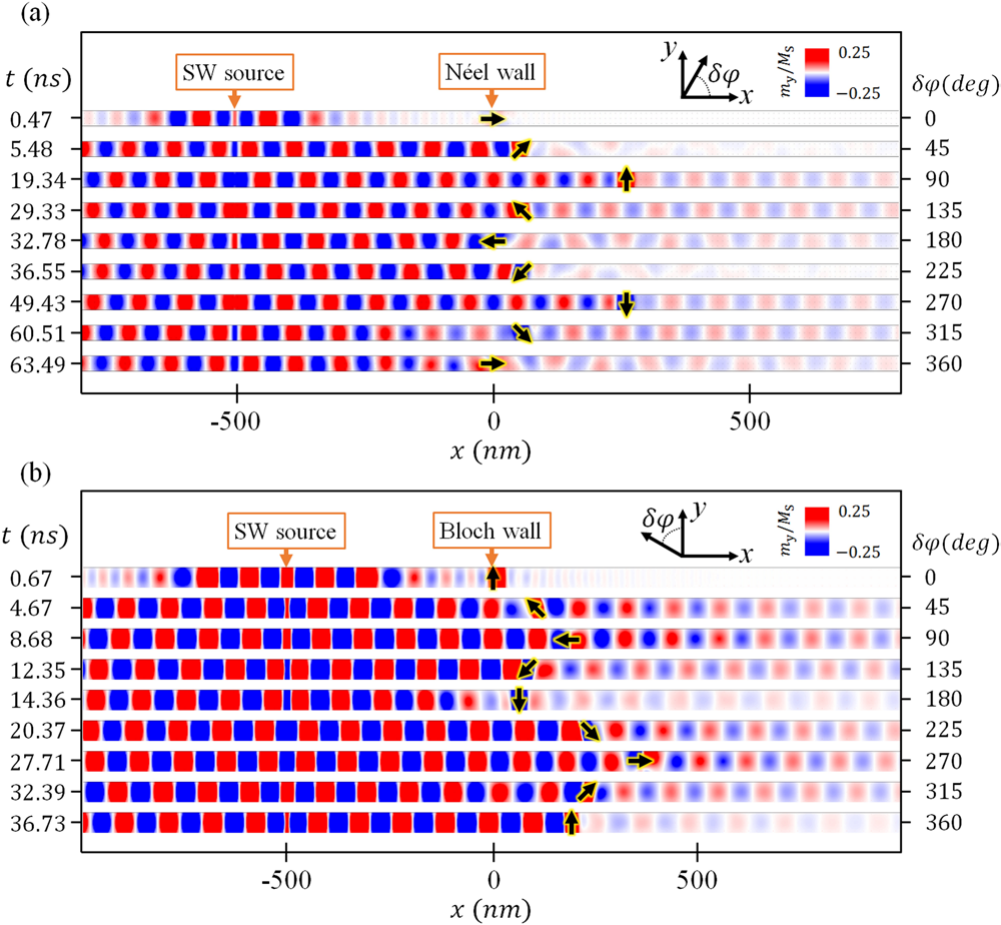
Time evolution of the y component of the magnetization and the DW motion in the case of (**a**) a Néel wall and (**b**) a Bloch wall for the SW with 20 GHz frequency. On the left is the simulation time of the snapshot. The black arrow indicate the orientation at the center of the DW, noted on the right.

Figure 4(a) shows the simulated magnetization configurations in the case of a Néel wall as in Fig. 3(d), with $f$ = 20 GHz, *H*_0_*/M*_*S*_ = 0.274. The localized oscillation motions of the DW induced by the magnonic STT of the SW and the torque of the effective field are accompanied by in-plane counterclockwise rotation of the magnetization inside the DW. Once rotated by 90°, the DW shows a backward motion towards the SW source. For the case of an initial Bloch wall, the results are shown in Fig. 4(b) with $f$ = 20 GHz, *H*_0_*/M*_*S*_ = 0.34 as in Fig. 3(h). As we inspect our simulation results in Fig. 3, we find that the instantaneous velocity of the DW is directly associated with *δφ*. When *H*_0_ is smaller than the Walker breakdown field, *H*_w_, DW moves forwards and *δφ* is between 0° to 90°. When *H*_0_ is larger than *H*_w_, *δφ* can exceed 90°, where the DW plane rotation causes the demagnetizing torque and instantaneous velocity to change direction. The backward motion corresponds to *δφ* between 90° and 180°. Whenever a DW oscillation occurs*, δφ* rotates 360°, in which from 0° to 90° and 180° to 270° the DW moves forwards; otherwise, it moves backwards.

As presented in the supplementary materials, we have formulated an equation to calculate the rotation at the center of the DW plane from the simulation results, and we have also performed self-consistency checks of the DW instantaneous velocities.

### Interplay between the transmission ratio and *φ*

**Figure 5:**
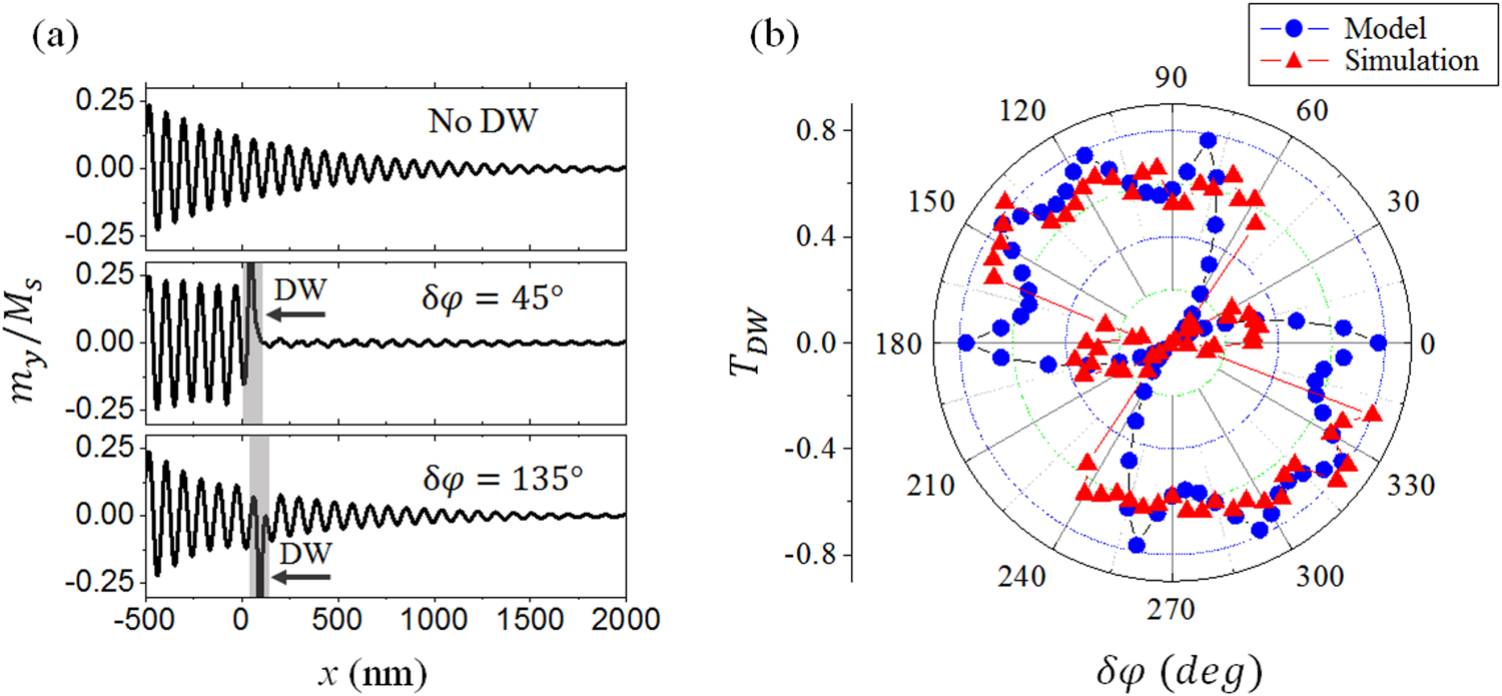
Transmission of SW with 20 GHz frequency as in the case of Fig. 3(d). (**a**) Spatial variation of the normalized *m*_*y*_ component without and with DW for *δφ* = 45° and 140°. Shaded areas indicate the domain wall region. (**b**) Polar plot of the transmission ratio versus *δφ*. Blue circles are calculated results and the red triangles are from simulations.

The SW transmission through a static DW with different angles of *φ* is presented in Fig. 2. When *H*_0_ is large and the DW is subject to motion, the SW transmission and *φ* are coupled as presented in the following observations. A spin wave is excited above the Walker breakdown threshold as illustrated in Fig. 3(c). Upon the incidence of the SW, the DW starts to move and the structure is modified. The first panel in Fig. 5(a) shows the spatial variation of the normalized *M*_y_ component without any DWs present while the SW propagates. The SW amplitude decays exponentially away from the source due to the damping in the material. The instantaneous magnetization of the small time varying component is given by ${\boldsymbol{m}}={{\boldsymbol{m}}}_{0}\,\exp \,[-i(kx-\omega t)]\,\exp \,(-x/{\rm{\Lambda }})$ where *m*_0_ is the SW amplitude at the source, and Λ is the attenuation length. In a uniform single domain, the attenuation length depends on α and the dispersion relation. In the presence of a DW, the effective anisotropy field exerts a torque to change *φ* when the SW travels in the DW, thus giving rise to a demagnetization field inside the DW region as ${\mathop{H}\limits^{\rightharpoonup }}_{d}=-{M}_{S}({N}_{x}cos{\rm{\phi }}\hat{x}+{N}_{y}sin{\rm{\phi }}\hat{y})$. The demagnetization factor *N*_*x*_ and *N*_*y*_ are determined by the DW width and the sample width, respectively. For very narrow wires, *N*_*y*_ may be close to unity. Taking this field into account, the attenuation length depends on the change of *φ*, *δφ*. As we show in the supplementary materials, the *T*_*DW*_, defined as the spin wave amplitude ratio with and without the DW on the +x side of the DW, can be written as

2 $${T}_{{\rm{DW}}}={e}^{-2{\rm{\Delta }}|\frac{1}{{{\rm{\Lambda }}}_{D}}-\frac{1}{{{\rm{\Lambda }}}_{0}}|},$$

with

3 $$\frac{1}{{{\rm{\Lambda }}}_{D}}=\frac{1}{d{e}^{-2kd}}[\frac{\alpha \gamma {M}_{s}}{\omega }({N}_{x}+{N}_{y}){N}_{x}{N}_{y}si{n}^{2}2\delta \phi +\alpha ({N}_{x}+{N}_{y})sin2\delta \phi ],$$

where Δ is the width of the DW, Λ_*D*_ is the attenuation length inside the DW, and Λ_0_ is the attenuation length outside the DW. When the SW propagates across the DW, we find *T*_*DW*_ is a function of *δφ*. Note that both *T*_*DW*_ and *δφ* are functions of the geometry and demagnetization factors, etc., of a nanostrip. The dependence of *T*_*DW*_ on *δφ* can be different for different nanostrips. Previous studies compared the SW wavelength and the DW width and found high *T*_*DW*_ for small SW wavelengths associated with backward DW motions^27,28^. Forward DW motion occurs at large wavelengths when *T*_*DW*_ is small due to the linear momentum transfer. This concept does not describe all the cases as we show here for only one SW frequency (wavelength). In Fig. 4(b), the Bloch wall moves forwards while *T*_*DW*_ increases from 0.03 at *δφ* = 0° (360°) to 0.6 at *δφ* = 90°. However, the Néel wall moves forwards from *δφ* = 0° to *δφ* = 90° as shown in Fig. 4(a) while *T*_*DW*_ first decreases then increases as shown in Fig. 5(b). We find that *δφ* is a decisive factor for the DW motion and the transmission of SWs, especially at low frequencies. The calculated *T*_*DW*_ for the case of Fig. 3(d) is plotted in Fig. 5(b) as blue circles. There are two minima at *δφ* = 45°, 225° and high, oscillatory values in the second and fourth quadrants. Here we have treated the DW rigidly with one single *φ*. Variation of *φ* inside a DW will make the analysis much more complicated.

The simulation results indeed show more interesting behavior. Here *T*_*DW*_ is defined as the SW amplitude at the position *x* = 0.5 µm divided by the amplitude without a DW present, regardless of the DW position. Figure 5(a) shows the snapshots of the SW amplitude (normalized *m*_*y*_ component) in the nanostrips without and with a DW present with selected values of *δφ*, 45° and 140°. The shaded areas indicate the position of the DWs. For the 45° DW *T*_*DW*_ = 0.016, the SW is almost unable to propagate across the DW and most of the SW is reflected. However, for the 140° DW, the SW can penetrate the DW with relatively high transmission, *T*_DW_ = 0.822. The red triangle symbols in Fig. 5(b) show the simulation results of DW angular dependence of *T*_DW_ for a propagating SW with *f* = 20 GHz and *H*_0_*/M*_*S*_ = 0.274. It is strongly anisotropic. The minimum *T*_DW_ occurs at −10°, 45°, 175°, and 225° when the DW speed or acceleration is maximum, please see supplementary materials. The transmitted SW shows a monotonically increasing phase shift^14,19^ when *δφ* increases and π phase jumps at *δφ* = 45° and 225°. The DW serves as a SW switch at relatively low frequencies and results in rich DW dynamics. Thus, *δφ* acts as an amplitude filter for SW propagation in the PMA nanostrips, and can be engineered to control SWs in practical devices. We could consider the DW-based magnonic waveguide as an information memory device where one can switch on or off the transmission of the SW through the device by manipulating the orientation of the DW without an external field or charge current.

In summary, we have found that the SW shows highly anisotropic transmission through different orientations of magnetization inside a DW at a relatively low frequency. When the SW amplitude is large, it induces an effective field torque leading to the rotation of the DW plane. The forward DW motion is a contribution of the demagnetization field due to the increase of the transverse components of magnetization in the DW region, and thus yields an increase of the magnetization orientation of *δφ*. The transmission ratios of the SW are in turn determined by *δφ* of the DW and show complicated dependence at low frequencies. We can thus manipulate the DW motion by selecting the SW frequency and/or controlling the SW amplitude through adjusting the DW angles. The interplay between the SW and the DW offers a wide range of possibilities for circuit design in spintronics.

## Methods

The PMA material we present here is NiFe^27,28,33^. The nanostrip studied is 4 μm long in the x direction, as shown in Fig. 1(a). The strip width w is from 10 to 80 nm and the thickness d is from 1 to 10 nm. To study the SW and DW dynamics, micromagnetic simulations have been performed with the micromagnetic code OOMMF^34^ with a unit cell of 2 × 2 × d nm^3^. The values of material parameters were saturation moments *M*_S_ = 8.6 × 10^5^ A/m, the exchange stiffness constants *A* = 1.3 × 10^−11^ J/m, the perpendicular anisotropy constants *K*_⊥_ = 5.8 × 10^5^ J/m^3^, and the damping parameter *α* = 0.01. To find the initial natural equilibrium state of the 180° DW structure, simulations starting from a nanostrip with opposite domain structures on both sides of a 20 nm wide central random state is relaxed to the stable DW state. The resulting structures of the DW are dependent on the width and thickness of the nanostrip. The DW at the center of the strip is subjected to a SW source located 500 nm on the left. The SWs are excited locally in an area 2 nm across, shown as the green part in Fig. 1(a), by a harmonic sinusoidal field ${\rm{H}}={{\rm{H}}}_{0}\,\sin (2{\rm{\pi }}\mathrm{ft}){{\rm{e}}}_{{\rm{y}}}$ with amplitude *H*_0_ in the transverse direction *y* and frequency *f*. The field amplitude *H*_0_ is in the unit of *M*_*S*_. There is no dc external magnetic field when the SW is active.

## Electronic supplementary material

Supplementary Materials
