# Supplementary material for: Ferromagnetic domain walls as spin wave filters and the interplay between domain walls and spin waves
Source: Sci Rep. 2018 Mar 2;8:3910. doi: 10.1038/s41598-018-22272-2 (PMC5834505; doi:10.1038/s41598-018-22272-2)
Supplement: Supplementary file 1 — Supplementary Materials [file 41598_2018_22272_MOESM1_ESM.docx]

**Supplementary Materials**

Ferromagnetic domain walls as spin wave filters and the interplay between domain walls and spin waves

Liang-Juan Chang^1^, Yen-Fu Liu^1^, Ming-Yi Kao^1^, Li-Zai Tsai^1^, Jun-Zhi Liang^2, *^, and Shang-Fan Lee^1, **^

^1^ Institute of Physics, Academia Sinica, Taipei 11529, Taiwan

^2^ Department of Physics, Fu Jen Catholic University, Taipei 24205, Taiwan

The rotation of the domain wall (DW) plane plays a crucial role in the DW dynamics. When a spin wave (SW) with a relatively low frequency *f* = 20 GHz and large enough amplitude *H_0_/M_S_* = 0.19 is applied, we observed from the simulation a forward DW motion, shown in Fig. 3(b) in the main article, and a rotation of the DW plane. The temporal evolution of the rotation at the center of the DW plane, *δϕ*, is shown in Fig. S1(a). While the DW is moving away from the source, the wall plane rotates in a close analogue to the slope of the DW displacement. In the initial state, the local magnetization ***M*** inside a 180°-domain-wall is located at *x* = 0, with azimuth angle *φ* = 0°, oriented as defined in Fig. 1. The dynamics of the interaction between the SW and the DW is described by the modified Landau-Lifshitz-Gilbert (LLG) equation [S1],

$\frac{\partial M}{\partial t}=-\gamma M\times H_{\mathrm{eff}}+\frac{\alpha}{M_{s}}M\times\frac{\partial M}{\partial t}-\frac{\partial J_{m}}{\partial x}$ (S1)


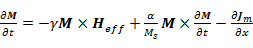


, where *γ* is the gyromagnetic ratio, *α* is the Gilbert damping parameter, *M*_s_ is the saturation magnetization, and ***H***_eff_ is the effective magnetic field consisting of anisotropy, demagnetization, and exchange fields, and where ***J***_m_ $J_{m}$is the magnon spin current. Hinzke *et al*. [S2] and Yan *et al*. [S3] have proposed a magnonic STT mechanism to explain the SW-induced wall motion. The DW must propagate in the opposite direction to that of the magnon with a velocity ***V***_m_$V_{m}$ to compensate the magnon spin current changes. This scenario is valid only when the wavelength is smaller than the DW size. As a SW passes through the DW, the total instantaneous magnetization can be expressed as the vector sum of a main equilibrium component and a small fluctuating time-varying component, ***M(x,t)*** = ***M***_0_ *+* ***m(x,t)*** *with* $\boldsymbol{m}\boldsymbol{(x,t)}=\boldsymbol{m}_{0}exp[-i(kx-\omega t)]exp(-x/\Lambda)$ in the Cartesian coordinates, where Λ is the characteristic attenuation length. In our case Λ ~ 370 nm. At the center of the DW, ***M***_0_ is perpendicular to the easy axis$\boldsymbol{e}_{z}$. Therefore, only the fluctuation component contributes to the effective anisotropy field ***h***_K_. In this case, $h_{K}={2K_{\perp}\delta m_{z}e_{z}}/{\mu_{0}M_{s}^{2}}$ [S4], where $\delta m_{z}=m_{z0}e^{-x/\Lambda},$and $m_{z0}$is the z component of the fluctuation at $x=0$(the initial position of the DW). As shown in Fig. S1, ***h***_K_ along $\boldsymbol{e}_{z}$ exerts a torque $\boldsymbol{\tau}_{K}$ that rotates the DW around the $\boldsymbol{e}_{z}$ axis, and raises *δφ*,


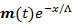

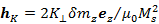

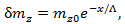


$\frac{d\delta\varphi\left( t \right)}{\mathrm{dt}}=$ $\gamma\left( h_{K}-h_{W}\sin2\delta\varphi\right)$, (S2)


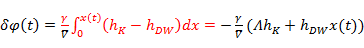


with $h_{W}$being the Walker field [S5].

When the ***h***_K_ exerts a torque to rotate the DW magnetization, an effective demagnetization field${\boldsymbol{h}_{\mathbf{d}}\mathbf{=-}N_{y}M_{y}\mathbf{e}}_{\mathbf{y}}$, where$N_{y}$is the demagnetization factor related to the DW itself, is simultaneously generated. The demagnetization torque$\tau_{d}=-\gamma M\times h_{d}$ cants the magnetization of the DW, resulting in the DW motion with the same direction of the SW, as shown in Fig. S1. The instantaneous DW velocity ***V***_d_ $V_{d}$induced by this demagnetization field in the presence of damping can be obtained using the rigid DW model [S6],


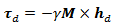


$V_{d}=-\frac{\gamma\Delta}{2\left( 1+\alpha^{2} \right)M_{S}}\left[ \left( M\times h_{d} \right)\cdot e_{z} \right]e_{x}$ (S3)


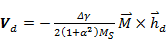


where Δ is the DW width. To estimate the total instantaneous velocity$\mathbf{V}_{\mathrm{DW}}=\mathbf{V}_{d}+\mathbf{V}_{m}$, we follow Ref. [S3] and obtain

$V_{\mathrm{DW}}=(\frac{\gamma\Delta M_{S}\left( N_{y-}N_{x} \right)}{2\left( 1+\alpha^{2} \right)}sin2\delta\varphi- \frac{\left[ e^{-\left( x-x_{0} \right)/\Lambda} \right]^{2}}{2}\frac{\partial\omega}{\partial k})$ $\boldsymbol{e}_{x}$ (S4)


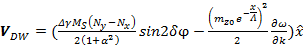


, where $\frac{\partial\omega}{\partial k}=2Ak$ is the SW group velocity, x_0_ is the location of the SW source. The rotation of the DW plane *δϕ*, the total instantaneous velocity **V**_DW_, and the DW acceleration are calculated and shown as red lines in Figs. S2(a) and (b), and (c), which show good agreements with the simulation results presented as circle, diamond, and triangle symbols, respectively. The magnitude of the **V**_DW_$V_{\mathrm{DW}}$ first increases as the *δϕ*increases, then the DW propagates away from the SW source, and the wall motion stops eventually because the magnitude of the effective anisotropy torque can no longer overcome the shape anisotropy effect. The maximum **V**_DW_ = 27 m/s$V_{\mathrm{DW}}=27m/s$ occurs around *δϕ =* 40°. At higher SW frequencies and for *H_0_* > *H_W_*$H_{0}>H_{W}$, as in Fig. 3(c), *δϕ* can reach 45° and the first term on the right of Eq. (S4) vanishes, thus the Walker breakdown occurs.


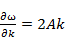

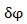

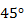


The total effective field for a Néel wall in Eq. (S1) is given by

$H_{eff}=H_{d}+h\left( x \right)e^{i\omega t}=(H_{dx}+h_{x}e^{i\omega t}, H_{dy}+h_{y}e^{i\omega t}, h_{z}e^{i\omega t})$, (S5)


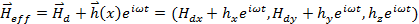


$H_{dx}=-M_{S}N_{x}cos\delta\varphi$ (S6)

$H_{dy}=-M_{S}N_{y}sin\delta\varphi$ (S7)

where $h(h\ll H_{d})$ is the r. f. exciting magnetic field with an angular frequency ω.


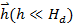


The magnetization at the center of the DW can be described by,

$M=(M_{x}+m_{x}e^{i\omega t}, M_{y}+m_{y}e^{i\omega t}, m_{z}e^{i\omega t})$ (S8)

$M_{x}=M_{S}cos\delta\varphi$ (S9)

$M_{y}=M_{S}sin\delta\varphi.$ (S10)

Inserting Eqs. (S5) – (S10) into the modified LLG equation (S1) and neglecting the higher order terms, we write the dynamic equations as

$\left[ \begin{matrix} i\omega& 0 & -\omega_{y} \\ 0 & i\omega& \omega_{x} \\ \omega_{y} & -\omega_{x} & i\omega\end{matrix} \right]\left[ \begin{matrix} m_{x} \\ m_{y} \\ m_{z} \end{matrix} \right]=-\gamma M_{s}\left[ \begin{matrix} -sin\delta\varphi h_{z} \\ cos\delta\varphi h_{z} \\ sin\delta\varphi h_{x}-cos\delta\varphi h_{y} \end{matrix} \right]$ . (S11)

Then,

$\left[ \begin{matrix} m_{x} \\ m_{y} \\ m_{z} \end{matrix} \right]=\frac{-i\gamma M_{s}}{\omega_{x}^{2}+\omega_{y}^{2}-\omega^{2}}\left[ \begin{matrix} \frac{-\omega^{2}+\omega_{x}^{2}}{\omega} & \frac{\omega_{x}\omega_{y}}{\omega} & {i\omega}_{y} \\ \frac{\omega_{x}\omega_{y}}{\omega} & \frac{-\omega^{2}+\omega_{y}^{2}}{\omega} & {-i\omega}_{x} \\ {-i\omega}_{y} & {i\omega}_{x} & -\omega\end{matrix} \right]\left[ \begin{matrix} -sin\delta\varphi h_{z} \\ cos\delta\varphi h_{z} \\ sin\delta\varphi h_{x}-cos\delta\varphi h_{y} \end{matrix} \right]$ (S12)

with

$\omega_{x}=\left( \gamma H_{dx}+\frac{\alpha}{M_{s}}i\omega M_{x} \right)$ (S13)

$\omega_{y}=\left( \gamma H_{\mathrm{dy}}+\frac{\alpha}{M_{s}}i\omega M_{y} \right)$. (S14)


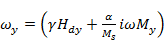


Inside the DW, we consider the dispersion relation of the magnetostatic surface spin wave (MSSW) [S7]

$\omega^{2}=\omega_{H}\left( \omega_{H}+\omega_{M} \right)+\frac{\omega_{M}^{2}}{2\left( 1+coth(kd) \right)}$ (S15)

with $\omega_{H}=\frac{\omega_{x}\omega_{y}}{\omega}$ and $\omega_{M}=\gamma M_{s}$. Using $k=k_{r}-i\kappa$ we get an identity

$\frac{1}{\left( 1+coth(kd) \right)}=\frac{1}{2}\left( 1-e^{-2k_{r}d} \right)+i\frac{d}{\Lambda}e^{-2k_{r}d}$ (S16)

with $\Lambda=1/\kappa$. After substituting *ω_H_* in terms of Eqs. (S13) and (S14), *ω_M_*, and Eq. (S16) into Eq. (S15), one obtains

$\frac{1}{\Lambda_{D}}=\frac{1}{de^{-2kd}}\left[ \frac{\alpha\gamma M_{s}}{\omega}\left( N_{x}+N_{y} \right)N_{x}N_{y}{sin}^{2}2\delta\varphi+\alpha\left( N_{x}+N_{y} \right)sin2\delta\varphi\right]$, (S17)

where Λ*_D_* is the attenuation length inside the DW. We find the *T_DW_*, defined as the spin wave amplitude ratio with and without DW on the +x side of DW, to be

$T_{DW}=e^{-2\Delta\left| \frac{1}{\Lambda_{D}} - \frac{1}{\Lambda_{0}} \right|},$ (S18)

where Δ is the width of the DW and Λ_0_ is the attenuation length outside the DW.

**Results for CoFeB**

The time evolution of the DW motion driven by SWs for the PMA materials of CoFeB is shown in Fig. S3. The strip width is 50 nm and thickness is 1 nm, following the experimental structure Ta(5 nm)/Co_20_Fe_60_B_20_(1 nm)/MgO(2 nm)/Ta(5 nm) [S8]. The values of material parameters for CoFeB were saturation moments *M*_S_ = 8.75 A/m, exchange stiffness constants *A* = 1.0 J/m, the perpendicular anisotropy constants *K*_⊥_ = 5.1 J/m^3^, and the damping parameter *α* = 0.01. For CoFeB with *f* = 5 GHz, the initial transient backward motion for all cases is associated with the separation between the DW and the SW source. When compared with the data of NiFe, the DW motion in CoFeB shows similar behaviors. As we describe in the main article, forward displacement after the initial transient motion was obtained in the wide range with small excitation amplitude shown as black line for H_0_ = 120 mT, red line for H_0_ = 130 mT, and blue line for 135 mT in Fig. S3(a). These motions are associated with changes of azimuthal angle, *δϕ*, at the center of the DW structure. CoFeB shows region I behavior when *H*_0_ ≤ 138 mT. The damped oscillatory motion of the DW is due to the relativity larger attenuation length of the propagation SWs in this material. CoFeB shows region II behavior when 139 mT ≤ *H_0_* ≤ 139.9 mT. We did not find localized oscillatory motion with 0.01 mT resolution between regions II and III. In region III, oscillatory motions of the DW associated with propagations in the opposite direction to the SW as shown by the pink line for *H_0_* =139.93 mT, green line for *H_0_* =140 mT, and purple line for *H_0_* =160 mT in Fig. S3.

**References:**

S1. Wang, X.-g., Guo, G.-h., Nie, Y.-z., Zhang, G.-f., and Li, Z.-x. Domain wall motion induced by the magnonic spin current. *Phys. Rev. B* **86,** 054445 (2012).

S2. Hinzke D. and Nowak, U. Domain wall motion by the magnonic spin Seebeck effect. *Phys. Rev. Lett.* **107,** 027205 (2011).

S3. Yan, P., Wang, X. S., and Wang, X. R. All-magnonic spin-transfer torque and domain wall propagation. *Phys. Rev. Lett.* **107,** 177207 (2011).

S4. Stancil D. D. and Prabhakar, A. *Spin Waves Theory and Applications* (Springer, New York, 2009).

S5. Mougin, A., Cormier, M., Adam, J. P., Metaxas P. J., and Ferré, J. Domain wall mobility, stability and Walker breakdown in magnetic nanowires. *Europhys. Lett.* **78,** 57007 (2007).

S6. Hillebrands B. and Thiaville, A. *Spin Dynamics in Confined Magnetic Structures* III (Springer, New York, 2006).

S7. Kaboš P. and Staĺmachov, V. S. *Magnetostatic Waves and their Applications* (Springer, Netherlands, 1994).

S8. Lo Conte, R. *et al*. Role of B diffusion in the interfacial Dzyaloshinskii-Moriya interaction in Ta/Co_20_Fe_60_B_20_/MgO nanowires. *Phys. Rev. B* **91,** 014433 (2015).


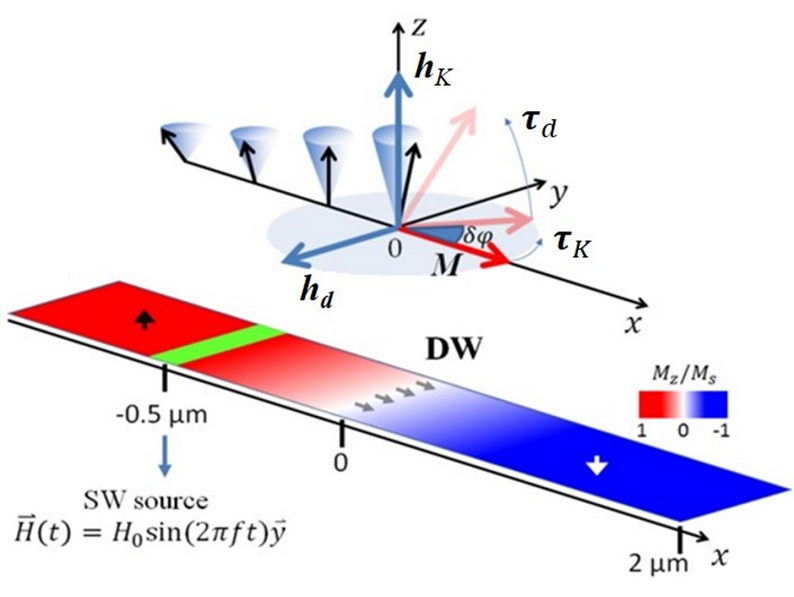


Fig. S1. The cones indicate the precession of the magnetizations. *δϕ* is the rotation of the magnetization at the center of the domain wall. ***τ****_d_*, ***h****_d_*, ***τ****_K_*, and ***h****_K_* are the torques and effective fields due to demagnetization and anisotropy, respectively. Drawing is not to scale.


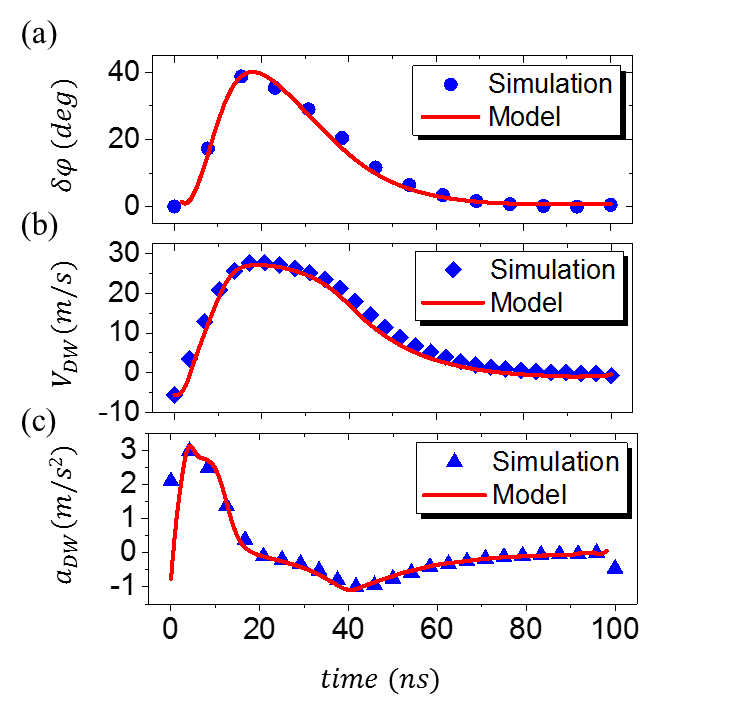


Fig. S2. (a) The rotation angle of the magnetization, *δϕ*, (b) instantaneous velocity, and (c) acceleration of the domain wall versus time for the case of Fig. 2 (b), SW frequency 20 GHz, amplitude *H_0_/M_S_* = 0.19.


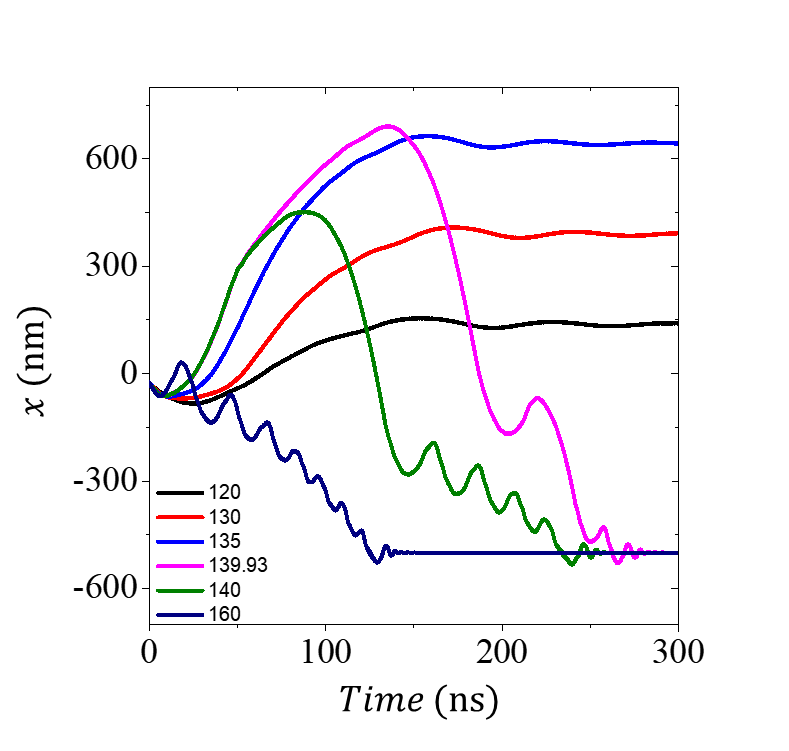


Fig. S3. Typical domain wall displacement as functions of simulation time and magnetic field amplitude of the spin wave with frequencies of 5 GHz for CoFeB.
